# Adrenal Tumors in Young Adults: Case Reports and Literature Review

**DOI:** 10.3390/medicina58060746

**Published:** 2022-05-30

**Authors:** Małgorzata Zdrojewska, Emilia Mech-Siebieszuk, Renata Świątkowska-Stodulska, Bartosz Regent, Michał Kunc, Łukasz Zdrojewski, Krzysztof Sworczak

**Affiliations:** 1 Department of Endocrinology and Internal Medicine, Faculty of Medicine, Medical University of Gdansk, 80-214 Gdansk, Poland; meszio@gumed.edu.pl (E.M.-S.); rensto@gumed.edu.pl (R.Ś.-S.); krzysztof.sworczak@gumed.edu.pl (K.S.); 2 Department of Radiology, Faculty of Medicine, Medical University of Gdansk, 80-214 Gdansk, Poland; bartoszregent@gmail.com; 3 Department of Pathology, Faculty of Medicine, Medical University of Gdansk, 80-214 Gdansk, Poland; michal.kunc@gumed.edu.pl; 4 Department of Nephrology, Transplantology and Internal Medicine, Faculty of Medicine, Medical University of Gdansk, 80-214 Gdansk, Poland; lukasz.zdrojewski@gumed.edu.pl

**Keywords:** adrenal tumor, pheochromocytoma, paraganglioma, endothelial cysts, lymphangioma

## Abstract

The current high detection rate of adrenal tumors (4–10% of general population) is attributable to a widespread use of variety of imaging studies, especially a computed tomography. Most of them represent clinically silent and biologically indolent incidentalomas, but some adrenal tumors may pose a significant clinical challenge. Thus, in every patient with an adrenal tumor, a decision on further management is made after careful hormonal and radiological evaluation. All hormonally active tumors and those with radiological features suggesting malignancy are qualified for surgery. Approximately 80% of adrenal tumors are adrenocortical adenomas, hypertrophy, or nodular adrenocortical hyperplasia. Other histopathological diagnoses include pheochromocytoma, adrenocortical carcinoma, metastases, mesenchymal tumors, lymphomas, cysts, and ganglioneuromas. Adrenal tumors are more commonly diagnosed and better studied in elderly patients. In younger patients, under 40 years old, focal adrenal lesions are relatively rare, and histological distribution of diagnoses differs from that in elderly individuals. Younger patients are more likely to display endocrine symptoms, which raise the suspicion of an adrenal mass. In the current study, we present a case series of seven adrenal tumors occurring in young patients. The cases presented below, along with the literature review, demonstrate that the diagnosis and treatment of adrenal tumors are crucial due to endocrinopathy-derived complications and a potential risk of malignancy.

## 1. Introduction

Adrenal tumors occur in approximately 4–10% of the general population, and their detection rate has increased with age [1,2,3,4] as a result of a widespread use of imaging studies. They are most often detected incidentally during imaging performed as a part of diagnostics of unrelated conditions. The peak incidence occurs in the seventh decade of life [4,5]. All patients found to have an adrenal tumor should undergo a detailed clinical, biochemical, and radiological evaluation to select those requiring surgical management [1,2,3,4].

The vast majority of adrenal tumors (approximately 80%) are adrenocortical adenomas, hypertrophy, or nodular adrenocortical hyperplasia. Other lesions include pheochromocytomas (PHEO) (3–6%), adrenocortical carcinomas (ACC) (2–5%), adrenal metastases (1–2%), mesenchymal tumors (myelolipoma, lipomas, hemangiomas, sarcomas), lymphomas, cysts, ganglioneuromas [1,2,3,4,6,7,8,9,10,11,12,13,14], other rare tumors [15], or collision tumors consisting of at least two different histopathological types [7,16].

This article presents seven cases of adrenal tumors detected in patients younger than 40 years old. In this age group, adrenal masses are relatively rare, and histological distribution differs from that in elderly individuals. They were divided into two groups—the first three cases concern hormonal active tumors, whereas the next four adrenal lesions did not show hormonal activity.

## 2. Materials

### 2.1. Case 1

**Figure 1:**
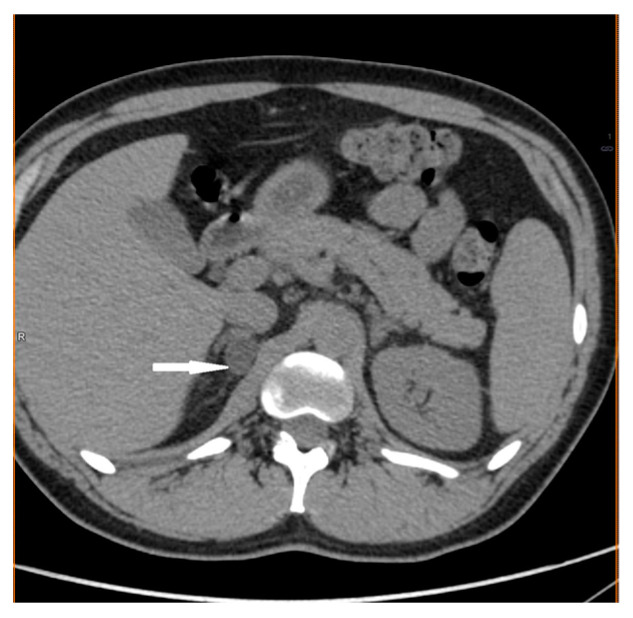
Adrenal cortical adenoma. Abdomen CT, transverse cross-section.

A 31-year-old man was admitted to hospital to investigate the cause of arterial hypertension that had occurred 1 year before, with peak values >200/100 mmHg and concomitant headache, reduced exercise tolerance, exertion dyspnea, and unintentional weight loss (6 kg) over past 3 months. On admission, he was in good general condition, with a BP of 170/100 mmHg. Physical examination revealed a systolic heart murmur and lower extremities edema. Biochemical tests showed hypokalemia (2.8 mmol/L) with normal sodium concentration (143 mmol/L) and proteinuria. Abdominal ultrasound (US), head computed tomography (CT), doppler ultrasound of carotid, vertebral, and renal arteries showed no clinically significant abnormalities. Transthoracic echocardiography (TTE) revealed left ventricular hypertrophy and left atrium enlargement. On fundoscopic examination, a third-grade hypertensive retinopathy in Keith–Wagner–Barker classification was diagnosed. An abdominal CT scan demonstrated low-density (–5 Hounsfield units, HU) right adrenal gland mass, measuring 15 × 11 × 19 mm with radiological features of adenoma, and thickening of the medial limb of the left adrenal gland up to 9 mm (Figure 1). In hormonal work-up, hypercortisolemia was excluded, and the 24 h urinary excretion of catecholamines metabolites was within the normal range (Table 1). However, increased plasma aldosterone concentration (54 ng/mL), decreased renin concentration (2.1 uIU/mL), plasma renin activity of 0.05 ng/mL/h, and aldosterone-to-renin ratio (ARR) of 1080 (normal ARR < 20) were found. An intravenous saline infusion test was not performed due to persistent hypokalemia resistant to potassium supplementation.

To confirm lateralization of aldosterone secretion, adrenal venous sampling (AVS) was performed, clearly indicating the right-sided aldosterone hypersecretion. A therapy with lercanidipine, ramipril, eplerenone, doxazosin, bisoprolol, and potassium was introduced, with effective control of BP and normokalemia. 

**Figure 2:**
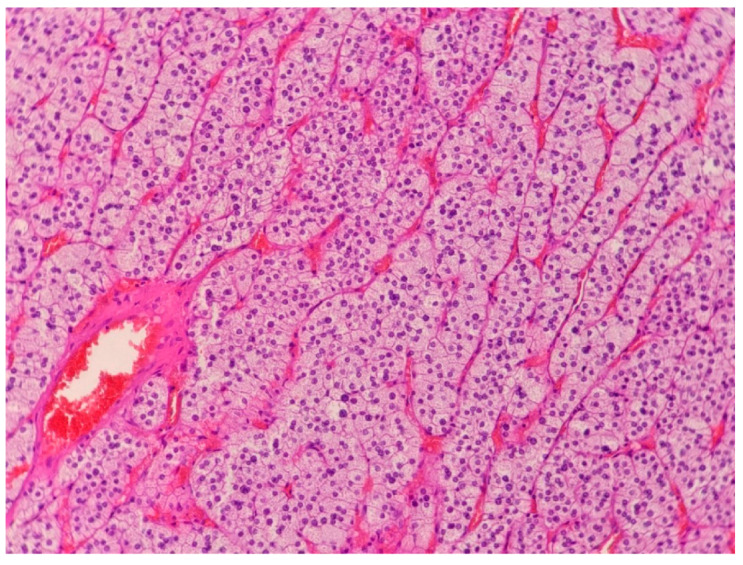
An adrenal cortical adenoma is composed of cells with a foamy cytoplasm, which is rich in lipids, and resembles cells of the zona fasciculata of the adrenal cortex.

The patient underwent right-sided laparoscopic adrenalectomy. The surgery resulted in the normalization of potassium levels and a significant improvement in BP control. Histopathologic examination of specimens showed an adrenocortical adenoma with moderate cellular pleomorphism, without mitotic activity, features of necrosis, or vascular invasion (Figure 2).

### 2.2. Case 2 

**Figure 3:**
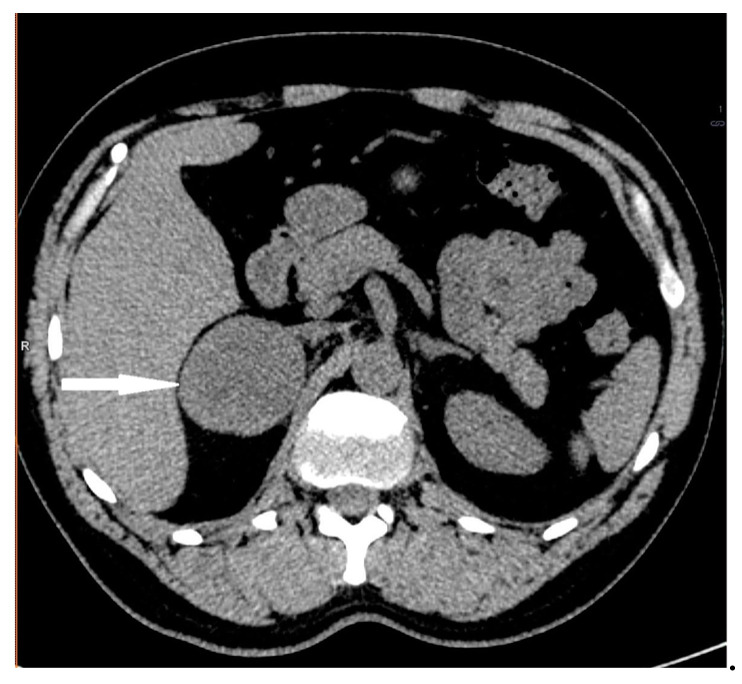
Pheochromocytoma. Abdomen CT, transverse cross-section.

A 34-year-old male was referred to hospital due to recently detected impaired renal function (serum creatinine 3.47 mg/dL, BUN 59.5 mg/dL, GFR 20 mL/min). His past medical history included hypertension (since the age of 24, poorly controlled with three antihypertensive agents—nebivolol, perindopril, amlodipine) and nocturia. On admission, increased BP (160/100 mmHg), obesity (Body Mass Index (BMI)—31 kg/m^2^), and liver enlargement were noted. A subsequent non-contrast (due to renal failure) CT scan revealed the presence of a mass originating from the right adrenal gland, measuring 56 × 58 × 56 mm (Figure 3). Based on hormonal tests, hypercortisolism was excluded, and secondary to renal failure, hyperaldosteronism was found. The concentration of urinary metanephrine was significantly increased (89961 μg/24 h, norm 64–302 μg/24 h) (Table 1). 

Due to poor BP control, as well as for clinical and radiological suspicion of PHEO, an alpha-blocker doxazosin was added to antihypertensive therapy in up-titrated doses to 16 mg per day. 

**Figure 4:**
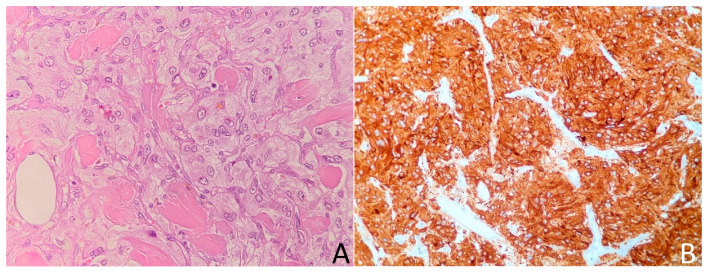
Pheochromocytoma—cells with moderate nuclear pleomorphism, prominent nucleoli, and abundant granular cytoplasm that form irregular nests. Hematoxylin & eosin, 400× magnification (**A**). Pheochromocytoma cells show strong cytoplasmic immunoreactivity for synaptophysin. 200× magnification (**B**).

Two months after the diagnosis, the patient underwent a right-sided laparoscopic adrenalectomy, which required a switch to open laparotomy due to intraoperative bleeding. Histopathological examination revealed PHEO. A single tumor embolus in a blood vessel was found, suggesting a malignant character of the lesion (Figure 4).

A CT scan performed two months after surgery showed no recurrence of the disease. Levels of urinary catecholamines were normal. Renal function did not improve significantly after surgery. The patient remained under nephrological care, and has recently received a pre-emptive kidney transplantation.

### 2.3. Case 3

A 37-year-old male patient was referred to the hospital for the diagnostics of poorly controlled arterial hypertension, diagnosed two years before. He was treated with two antihypertensive drugs (telmisartan, lercanidipine), with office BP values of 140/100 mmHg. Family history revealed that the patient’s mother was diagnosed with Cushing’s syndrome. 

The patient was obese (BMI 36 kg/m^2^), but otherwise, no signs of endocrinopathy were noted. CT scan detected a right adrenal mass measuring 36 × 29 mm with radiologic features of adenoma. Hormonal work-up revealed hypercortisolism (Table 1) with low morning ACTH concentration (6 pg/mL), and androstenedione and DHEA-S levels were below the reference range. Hypercholesterolemia, as well as biochemical and ultrasound markers of liver damage, were also found.

The patient was diagnosed with subclinical hypercortisolemia. In hormonal reassessment after four months, biochemical markers of hypercortisolemia persisted, and the low dose dexamethasone suppression test (Liddle’s test) revealed no suppression of cortisol secretion. The patient was subjected to an adrenalectomy. He did not require preoperative treatment with steroidogenesis inhibitors. In histopathological examination, an adrenal cortical adenoma was diagnosed.

### 2.4. Case 4

**Figure 5:**
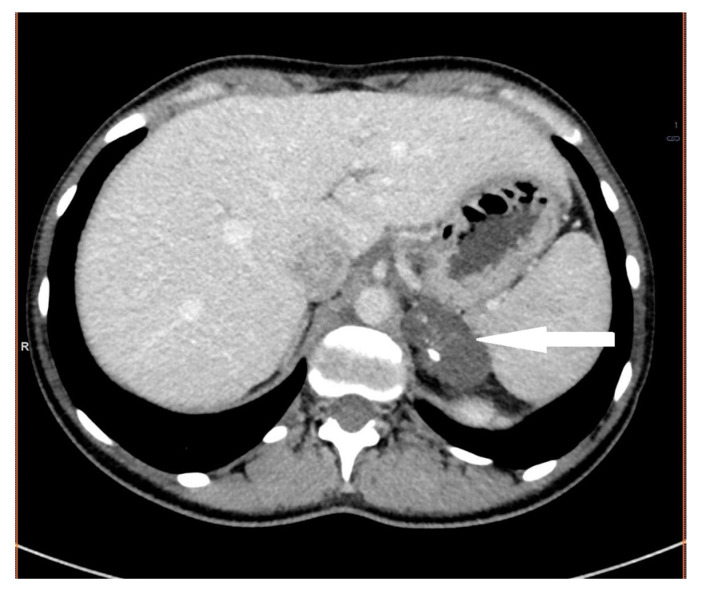
Endothelial cyst. Abdomen CT, transverse cross-section.

**Figure 6:**
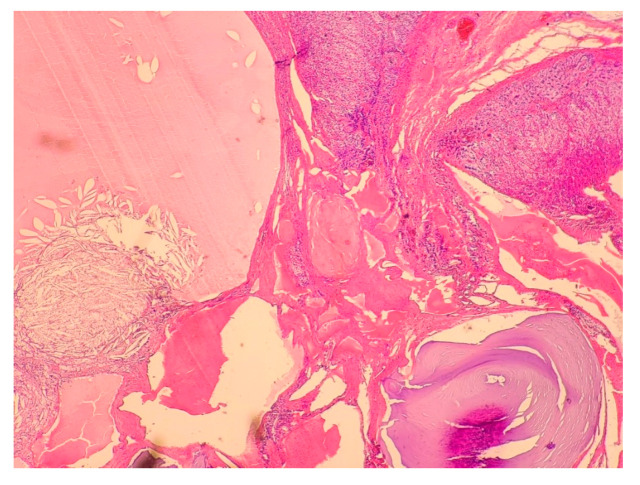
Multilocular endothelial cyst lined by endothelial cells with septal calcifications. In the lumen of the cyst, eosinophilic masses and cholesterol clefts are visible. The lesion probably represents dilated blood vessels with organized thrombi. Hematoxylin & eosin, 40× magnification.

A 27-year-old woman was admitted to the department of endocrinology clinic due to a left adrenal gland tumor detected in an abdominal ultrasound. The examination was performed as a part of diagnostics process of abdominal pain and unintentional weight loss (approx. 10 kg) over the past year. A physical examination revealed the patient to be underweight (BMI—17 kg/m^2^), with hemiparesis resulting from cerebral palsy, and tenderness on palpation of the umbilical and left hypochondriac region. Biochemical tests showed no significant abnormalities in hormonal work-up (Table 1). The CT scans demonstrated the presence of a polycyclic thin-walled cystic tumor in the left adrenal gland measuring 45 × 28 × 29 mm (Figure 5). The patient was referred for laparoscopic adrenalectomy. Histopathological examination revealed a multilocular cyst lined with attenuated endothelial cells with septal calcifications. The histological appearance corresponded to the diagnosis of endothelial cysts (EC) (Figure 6).

### 2.5. Case 5

**Figure 7:**
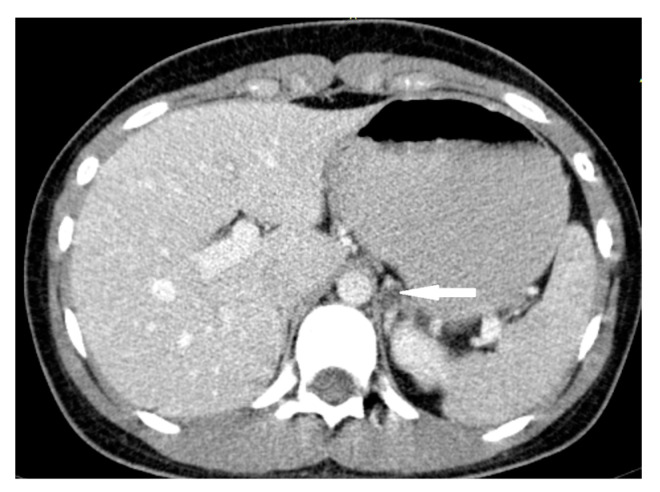
Adrenal lymphangioma. Abdomen CT, transverse cross-section.

**Figure 8:**
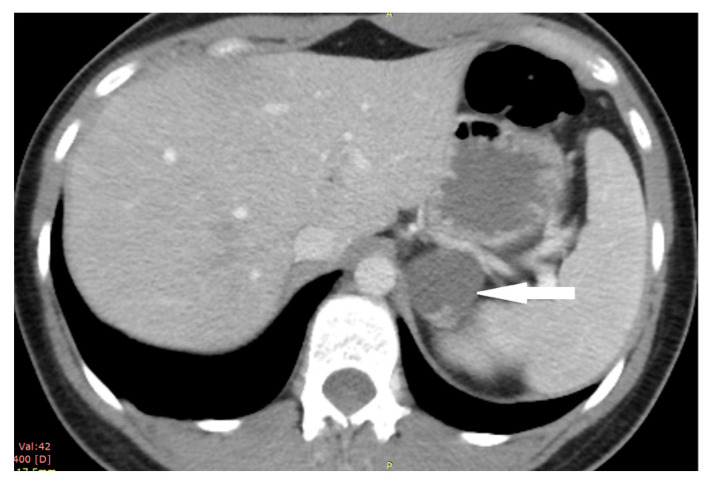
Adrenal lymphangioma. Abdomen CT, transverse cross-section, after eight years.

**Figure 9:**
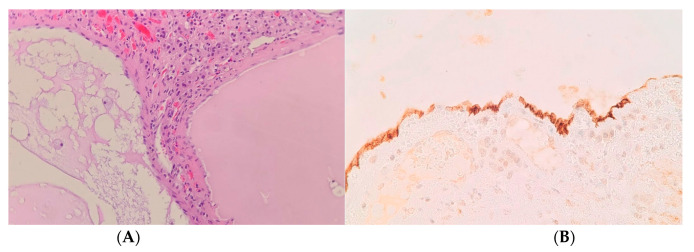
Adrenal lymphangioma—the tumor adjoins the adrenal cortex and consists of numerous cysts filled with proteinaceous content ((**A**), hematoxylin and eosin, 100× magnification), and lined with podoplanin-expressing lymphatic endothelium. Immunohistochemistry for podoplanin, 400× magnification (**B**).

A 19-year-old female patient was admitted to the department of endocrinology for hormonal evaluation, in the course of which a left adrenal gland mass was detected during the diagnostics of hyperandrogenism. The patient had a medical history of oligomenorrhea since puberty, as well as elevated serum testosterone and androstenedione concentration. The abdominal CT scan demonstrated a hypodense cystic lesion in the left adrenal gland, measuring 12 × 9 mm (Figure 7). The patient had mild hirsutism (assessed at three points on the Ferriman–Gallwey scale), but no other signs of endocrinopathy were noted. Biochemical tests revealed a slightly elevated concentration of androstenedione (3.8 ng/mL) and testosterone (4.91 nmol/L) (Table 1. P5 F/19). Due to clinical and laboratory features of hyperandrogenemia, a dexamethasone androgen-suppression test (5 days, 4 × 0.5 mg/d) was performed, resulting in the suppression of androgens concentrations. Based on these findings, a hormonally inactive adrenal tumor and functional hyperandrogenism were diagnosed. A CT scan performed eight years later revealed progression in size of the adrenal mass to 29 × 23 × 22 mm, with a solid part within it (Figure 8). Hormonal reassessment showed androstenedione and DHEA-S within the normal range of concentration (Table 1. P5 F/27). Due to the young age of the patient, the declared carcinophobia, and the progression in tumor size, a left-sided adrenalectomy was scheduled. Based on the histopathological examination of the adrenal gland, a diagnosis of lymphangioma was established (AGL) (Figure 9).

### 2.6. Case 6

**Figure 10:**
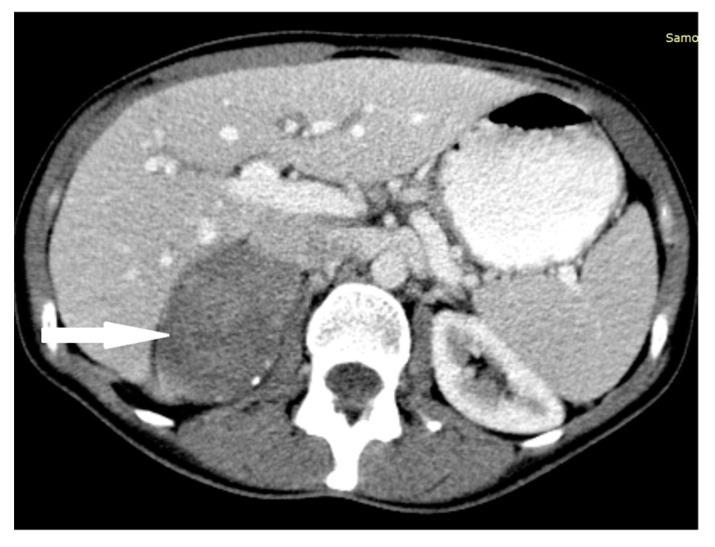
Ganglioneuroma. Abdomen CT, transverse cross-section.

**Figure 11:**
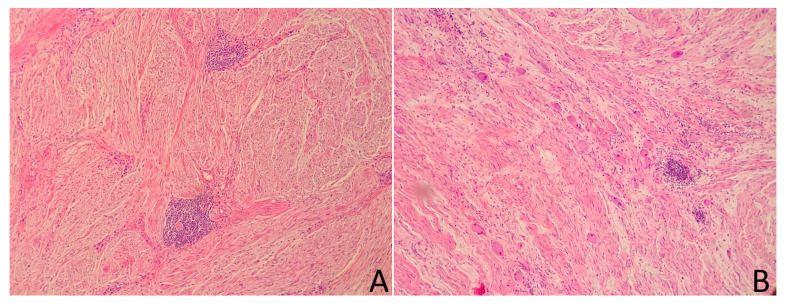
Ganglioneuroma—the tumor is mainly composed of Schwann cells forming fascicles in the myxoid stroma ((**A**), hematoxylin and eosin, 400× magnification). The mature ganglion cells are scattered throughout the tumor in small groups; they are characterized by abundant, eosinophilic cytoplasm and a round, eccentrically located nuclei ((**B**), hematoxylin and eosin, 400× magnification). Furthermore, focal lymphocytic infiltrates are visible (well visible in photo (**A**)).

A 37-year-old woman was referred to the hospital due to a right adrenal gland mass which was found in an ultrasonography carried out as a part of the diagnostics of non-specific abdominal pain. The CT scan confirmed the presence of an oval, well-demarcated lesion measuring 70 × 56 × 60 mm with peripheral calcifications and high density in the native phase (approximately 27–28 HU) (Figure 10). After administration of intravenous contrast, the central part of the mass was enhanced intensively and homogeneously. Radiological characteristics suggested a lesion other than adenoma. The patient did not report any symptoms typical for PHEO (blood pressure was normal, and her weight was stable). On general examination, no signs of endocrinopathy were found. Hormonal levels were normal (Table 1). Due to the ambiguous radiologic features of the tumor, she was subjected to laparoscopic adrenalectomy. Based on histopathological examination, a ganglioneuroma was diagnosed (GN) (Figure 11).

### 2.7. Case 7

**Figure 12:**
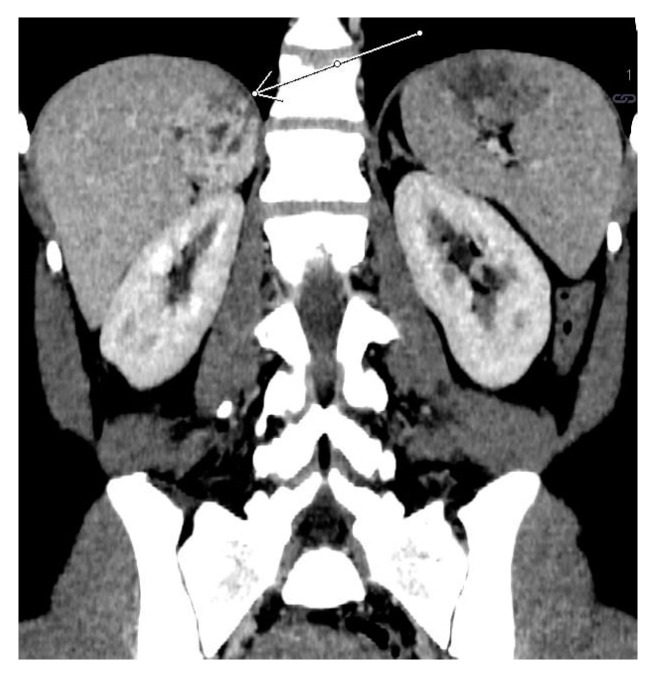
Adrenal cortical carcinoma. Abdomen CT, longitudinal cross-section.

A 33-year-old woman was referred to the endocrinology department due to a right adrenal mass. It was detected in ultrasonography performed due to dyspeptic symptoms. A tumor was described as a solid, heterogeneous, highly vascularized mass, merging with the liver capsule, and measuring 42 × 33 × 49 mm. Moreover, enlarged lymph nodes of the hepatic hilum were identified. The CT scan confirmed the presence of a right adrenal gland tumor with radiological features suggestive of malignancy or PHEO (due to the lesion’s native density of 21 HU, absolute percentage washout of 38%, and relative percentage washout of 32% 10 min after i.v contrast administration) (Figure 12).

**Figure 13:**
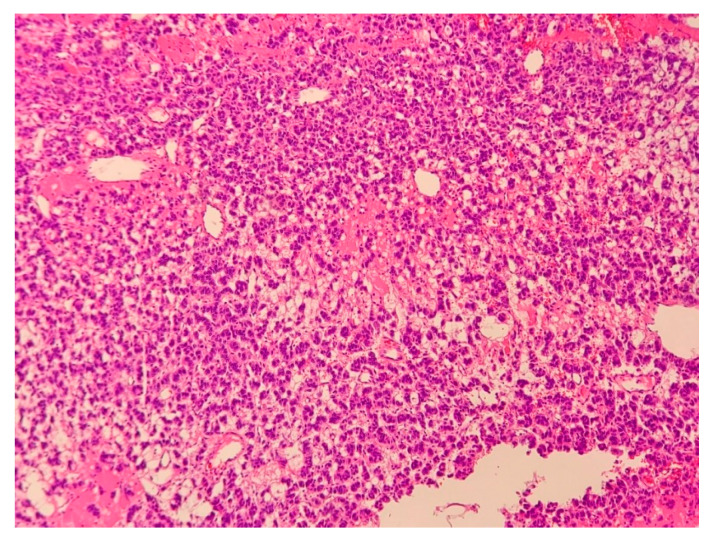
This adrenal tumor is composed of less than 25% of clear cells. Cells show moderate nuclear pleomorphism. A total of two blood vessels containing tumor emboli were found at the periphery of the tumor. According to the modified Weiss criteria, it suggests a malignant lesion. Hematoxylin and eosin, 40× magnification.

On admission, the physical examination, as well as hormonal evaluation, revealed no clinically significant abnormalities (Table 1). Due to ambiguous radiologic characteristics, the patient qualified for a unilateral adrenalectomy. Based on the postoperative histopathological examination of specimens, an adrenocortical carcinoma (ACC) was diagnosed (Weiss score of 3 points) (Figure 13). The imaging examinations during the five-year follow-up showed no signs of recurrence.

## 3. Discussion

The increasing detectability of adrenal tumors resulting from the widespread use of imaging studies allows us to make decisions regarding further management. The decision about follow-up or surgical intervention is often preceded by multidisciplinary examinations, carried out in accordance with the current guidelines [1,2]. The hormonal evaluation is intended to identify functional adrenal tumors. Routine tests for adrenocortical activity are performed to detect hypercortisolemia (serum cortisol circadian rhythm, 24 h urinary cortisol excretion, cortisol in overnight 1 mg dexamethasone suppression test, morning ACTH concentration) and hyperandrogenism (DHEA-S and androstenedione concentration). Adrenal medulla function is evaluated by determining the 24 h urinary excretion of catecholamines metabolites or plasma catecholamines, and these tests are used to diagnose PHEO. In special cases, diagnostics of primary hyperaldosteronism are carried out by determining the aldosterone to renin ratio (ARR), and by performing dynamic tests. Adrenal vein sampling is recommended to confirm primary hyperaldosteronism prior to the final qualification for adrenalectomy [1,2,4,17,18,19]. After hormonal evaluation, around 75–80% of tumors turn out to be nonfunctional lesions [1,2,6].

Imaging studies of adrenal tumors are assessed to distinguish between benign and malignant lesions [1,2,3,4,5,6,7,8,9,10,11,12,13,14,15,16,17,18,19,20,21]. The radiologic protocols allow for the determination of adenomas with a high probability (in CT evaluation of initial density, tumor’s morphology, a profile of contrast medium washout). On this basis, it is also possible to select tumors that are suspected to be malignant (primary or metastatic tumors). In borderline cases, the use of abdominal MRI allows for the recognition of lipid-poor adenomas and myelolipomas [22].

The diagnosis of PHEO, especially in mildly symptomatic or non-secreting forms, remains a diagnostic challenge [23]. If a malignant lesion is suspected, performing 18F-FDG PET/CT should be considered [2,20,22].

Eventually, adrenalectomy is recommended for hormonally active lesions, tumors with radiologic features described in imaging studies as “non-adenoma”, tumors with a diameter >3–6 cm [24,25,26], or tumors with rapid growth [1]. Due to relatively high percentage of malignant lesions in patients <40 years of age [2,6], some authors recommend adrenalectomy for tumors >3 cm in diameter, whereas for older patients, a tumor size >5 cm was adopted as a criterion [24]. The size criteria result from a higher frequency of ACC diagnosis in lesions larger than 4 cm [1,22]. Among the histological diagnoses, the most common are adenomas, hypertrophy, or nodular adrenocortical hyperplasia [1,2,6]. These lesions may be non-secreting, or they may present clinical syndromes in the form of hypercortisolemia, primary hyperaldosteronism, and hyperandrogenism [1,2,4,22]. The first two syndromes are often associated with an increased risk of complications, especially cardiovascular complications [5].

Adrenal cortical carcinoma is the primary tumor of adrenal glands with the worst prognosis. It affects females more frequently and displays two peaks of incidence in the first and fifth decades of life. It remains asymptomatic for a long time, so it is often diagnosed at the locally advanced or generalized disease stage. Despite a better understanding of the molecular pathology of ACC, and improved diagnostics and treatment, it still has high mortality rate [27,28,29].

The most common lesion originating from the adrenal medulla is PHEO, which accounts for approximately 3–6% of diagnoses [1,2,6]. Clinical symptoms, such as increased blood pressure, palpitations, headache, sweating, and tremors, may be paroxysmal [30]. They are characteristic; however in some cases may not occur [31]. Due to possible complications, especially caused by hypertension, the suspicion of PHEO is an indication for adrenalectomy preceded by α₁-adrenoreceptor antagonist preparation. In 10–15% of cases, an aggressive clinical course of PHEO is observed [32].

Adrenal metastases are common. Adrenal glands constitute the fourth most common site after lungs, liver, and bones of metastatic disease [33]. In histopathological examination, they are diagnosed with a frequency of 1–2%, and usually originate from lung, kidney, breast, and colon cancer, or melanoma [1,33,34].

Cysts are rare lesions of the adrenal glands. The incidence of adrenal cysts is below 0.2%, and shows a predilection for women [18]. They can be divided into pseudocysts (39%), endothelial cysts (45%), epithelial cysts (9%), or parasitic cysts (7%) [9,11]. Among endothelial cysts are lymphangiomas, which constitute 0.06% of all adrenal tumors. They can occur at any age, with a peak incidence between the third and sixth decades of life. Adrenal cysts are characterized by low density in CT imaging (usually <10 HU), as well as no enhancement after intravenous administration of a contrast agent. On MRI, they have low signal intensity on T1-weighted images and high signal intensity on T2-weighted images. Although CT and MRI can successfully determine the character of the tumor, these tests are usually not sufficient to make a final diagnosis. It should be emphasized that occasionally PHEO, adrenal metastases, and primary adrenal lymphoma may appear as cystic lesions, and, thus, should be considered in differential diagnosis [9,10,11]. 

Ganglioneuroma (GN) is another rare adrenal gland tumor. The prevalence of adrenal GN is estimated at 0.3–2%. GN is a benign, well-differentiated neurogenic tumor that arises from neural crest cells, and it is composed of intermixed Schwann cells, ganglion cells, and nerve fibers [14]. Ganglioneuromas are usually observed in the posterior mediastinum (39–43%), the retroperitoneal space (32–52%), and the cervical region (8–9%) [12]. Adrenal cysts and ganglioneuromas are hormonally silent in most cases, and remain asymptomatic throughout life. They are detected incidentally during imaging or because of nonspecific symptoms, such as abdominal discomfort or gastrointestinal symptoms. Laboratory tests are usually not helpful as a diagnostic tool, and diagnosis of both adrenal cysts and GN can be established through histological examination.

## 4. Conclusions

The prevalence of adrenal tumors increases with age, and, therefore, the elderly are most often hospitalized in endocrine departments. The majority of these patients are those with adrenal tumors which are found incidentally in image studies. Image studies, especially with the use of ionizing radiation techniques, are performed in younger patients only in the case of reported symptoms. 

Presented cases of adrenal tumors in the group of patients under 40 years of age describe both hormonally active and nonfunctional lesions. They can cause clinical symptoms and lead to serious complications. An active search for adrenal gland pathologies in that patient group has an important diagnostic and therapeutic significance. We should not underestimate adrenal tumors found in image studies, since, in this age group, diagnoses of ACC occur more frequently [2,6,27]. Most of the presented nonfunctional tumors, with radiological features that were not typical for adenoma, turned out to be benign. It seems that some of the adrenalectomies could have been avoided. However, the radiological appearance of these lesions was difficult to interpret. It likely results from the lack of unequivocal radiological criteria for these uncommon adrenal tumors. In ambiguous cases, 18F-FDG PET/CT should be considered to improve the diagnostic process.

## Figures and Tables

**Table 1:** Patient’s hormonal activity characteristics.

Patient Sex/Age	Cortisol Urinary 24-h Excretion (nmol/24 h)	ACTH Serum (pg/mL)	Cortisol Serum 1 mg DXM Suppression Test (nmol/L)	DHEA-S Serum (µg/dL)	Androstenedione Serum (ng/mL)	Metanephrines/Normetanephrines/3-Metoksythyramine Urinary 24 h Excretion (µg/24 h)
P1 M/31	-	7.8	<28	242	3.6	236/220/133
P2 M/34	144.0	31.3	-	306	2.8	89961/229/19
P3 M/37	798.0	6	169	77.1	<0.30	254/327/234
P4 F/27	212.0	8.5	<28	248	2.2	154/300/345
P5 F/19	100	32.2	<28	366	3.8	98/220/-
P5 F/27	87.4	22.3	<28	233	3.5	-/-/-
P6 F/37	141.0	19.4	<28	165	2.31	129/188/461
P7 F/33	288.0	15.5	<28	120	2.6	113/212/298

Normal ranges: 24-h cortisol urinary excretion: 12–486 nmol/24 h; serum ACTH: <46 pg/mL; serum DHEA-S males: 120 to 520 µg/dL; serum DHEA-S females: 65–380 µg/dL; 1 mg dexamethasone suppression test: <50 nmol/L; serum androstenedione males: 0.7–3.6 ng/mL; serum androstenedione males: 0.3–3.5 ng/mL; 24-h metanephrines excretion: 64–302 µg/24 h; 24-h normetanephrines urinary excretion: 162–527 µg/24 h; 24-h 3-metoxythyramine: 103–434 µg/24 h.

## Data Availability

Not applicable.
